# A New Method of Operating for the Restoration of the Female Perineum

**Published:** 1889-05

**Authors:** Hal C. Wyman


### A NEW METHOD OF OPERATING FOR THE RESTORA TION OF THE FEMALE PERINEUM.

BY HAL C. WYMAN, M. D., M. S.

Some years ago the writer published an account of a method for closing the perineum, and restoring the perineal body, in cases of sub-involution, without the removal of any tissue. The cases reported at that time have all progressed favorably. Since then the operation has been performed many times, and with such uniform success that I deem it no more than proper to speak of it again.

The operation, for purpose of description, may be divided into three stages. The first stage consists in introducing the finger into the rectum, pushing the recto-vaginal septum through the vulva and making a transverse incision about three-fourths of an inch in length through the mucous membrane near where it blends with the integument, or within the mucous membrane near where it blends with the integument, or within the mucous membrane of the rectum in cases of complete laceration of the perineum.

The second stage consists in dissecting through this incision the vaginal mucous membrane, from the recto-vaginal septum.

The third stage consists in closing the wound by carrying sutures across the denuded area, after introducing them a full half inch back of either angle of the transverse incision. The details of these stages are not difficult of execution. Sometimes the septum is so thin that one hesitates to attempt to dissect the vaginal mucous membrane off from it. In those cases the incision may be made to traverse the tissue a little higher up the rectum where it is much thicker. Some cases of complete laceration of the perineum, which had failed to unite after other operative methods, were successfully treated in this way. Not a particle of tissue being removed from the patient.

The second stage of the operation is to be accomplished mainly with the finger and blunt-pointed scissors. The operator keeps the recto-vaginal septum tense between the left index digit in the rectum, then with his right index he crowds up the vaginal mucous membrane, or with the blunt scissors cuts or breaks the connective tissue which binds it to the septum. In some cases I have found it necessary to dissect up in this way the entire inferior half of the posterior vaginal wall. I have never seen any serious hemorrhage nor have I known the parts to slough after such extensive separation.

I have operated in cases when the septum seemed too thin to admit of anatomical division. Sometimes the dissection is facilitated by using a blunt instrument like a lithotomy guide to hold the mucous membrane up against the anterior vaginal wall, as it is removed from the septum. The introduction of the suture must be guided by the character of the case. If there has been a complete laceration of the perineum the suture must be introduced with a long curved needle. It must begin about one half inch from the verge of the anus in its transverse meridian on the left side and must pass upward around the wound in the recto-vaginal septum emerging at the same point on the right side of the anus. When this suture is tied the inferior border of the transverse incision must be defended with a knot on an angle so that the knot comes across it. The second suture is introduced in the same way, only it begins about one-half inch higher on the border of the perineum and emerges on the side opposite. When it is tied the knot curves the wound, which, while it was originally transverse is now perpendicular and corresponds as the suture progresses to the raphe of the perineum. A third suture after this manner usually finishes the operation, but some times other shallower sutures are introduced to further close the wound. For this method of operating on the perineum when it is lacerated or subinvoluted I claim the advantages of, (1) easy and speedy execution; (2), a minimum of hemorrhage; (3), greater certainty of union, because of live tension on sutures; (4), a natural and perfect perineal body; (5), its adaptability to a wider range of cases than other operations.

DISCUSSION.

Dr. Manton: This operation was first done by Prof. Voss, Christiana, Norway, in 1865, but was suggested in 1862 by Prof. S. A. V. Stein, of Copenhagen. The operation as advocated by Voss, Tait, Jenks, and the writer of the paper, is substantially the same. Dr. Jenks lays great stress on taking deep stitches for the purpose of restoring the deeper portion of the perineal floor. It answers well in a certain portion of cases, but this specification which Dr. Jenks makes is important. The older writers have insisted that the several portions of the *transvers pereinei* should be brought together, but I believe that it is the *levator ani* which it is important to restore. We have often failed to get the result that we hoped for by the old operation.

Dr. Sprague: I have now done two operations by the method advocated in the paper, but one is too recent to report results and the other was not a favorable one for a test, being in an old lady and done to aid in correcting a serious cystocele. However, I am satisfied that this method has important advantages over the method of removing the membrane from the denuded surface, all that are claimed by the paper and, two advantages which I deem still more important, viz., protection of the healing surface from vaginal secretions and increased amount of tissue in the perineal support afforded by the membrane so left. So thoroughly am I satisfied of these advantages that I propose to operate by this method in the future.

Dr. Wyman: I wish to state some points of interest relating to the history. I am not so well read in gynecological literature as Dr. Manton, but after reading an article by Dr. Marion Sims on the subject some years since I conceived that the irritation and vaginismus which resulted in some cases might be avoided if the flap were left. Accordingly I operated so about 1880 and reported my results in some of the local journals. I do not claim to have originated the operation, but I supposed when I first performed it, that it was original. I consider this operation specially indicated in those cases where the *levator ani* is torn without much external laceration. I was at first puzzled to find all the symptoms of lacerated perineum present in cases where the external perineum was nearly entire, but have found as much benefit from the operation in these cases as in any.—*American Lancet.*

